# The prognostic impact of neoadjuvant chemoradiotherapy on lymph node sampling in patients with locally advanced rectal cancer

**DOI:** 10.1007/s13304-020-00841-3

**Published:** 2020-07-06

**Authors:** Giovanni Li Destri, Andrea Maugeri, Alice Ramistella, Gaetano La Greca, Pietro Conti, Giovanni Trombatore, Giada Maria Vecchio, Gaetano Giuseppe Magro, Martina Barchitta, Antonella Agodi

**Affiliations:** 1 Department of Medical and Surgical Sciences and Advanced Technology “G.F. Ingrassia”, University of Catania, Via Santa Sofia 86, Catania, Sicily Italy; 2 U.O.C. General Surgery, ASP Siracusa, Contrada Colle Roggio, Lentini, Sicily Italy

**Keywords:** Rectal cancer, Lymph node sampling, Neoadjuvant chemoradiotherapy, Lymph node ratio

## Abstract

According to the American Joint Committee on Cancer, at least 12 lymph nodes are required to accurately stage locally advanced rectal cancer (LARC). Neoadjuvant chemoradiation therapy (NACRT) reduces the number of lymph nodes retrieved during surgery. In this study, we evaluated the effect of NACRT on lymph node retrieval and prognosis in patients with LARC. We performed an observational study of 142 patients with LARC. Although our analysis was retrospective, data were collected prospectively. Half the patients were treated with NACRT and total mesorectal excision (TME) and the other half underwent TME only. The number of lymph nodes retrieved and the number of metastatic lymph nodes were significantly reduced in the NACRT group (*P* > 0.001). In the univariate and multivariate analyses, only NACRT and patient age were significantly associated with reduced lymph node retrieval. The number of metastatic lymph nodes and the lymph node ratio (LNR) both had a significant effect on prognosis when the patient population was examined as a whole (*P* = 0.003 and *P* = 0.001, respectively). However, the LNR was the only significant, independent prognostic factor in both treatment groups (*P* = 0.007 for the NACRT group; *P* = 0.04 for the no-NACRT group). NACRT improves patient prognosis only when the number of metastatic lymph nodes is reduced. The number of metastatic lymph nodes and the LNR are important prognostic factors. Lymph node retrieval remains an indispensable tool for staging and prognostic assessment of patients with rectal carcinoma treated with NACRT.

## Introduction

In Western countries, colorectal carcinoma is one of the main causes of cancer-related death. Approximately, 75% of patients with colorectal cancer undergo radical treatment [1]. Neoadjuvant chemoradiation therapy (NACRT) followed by total mesorectal excision (TME) has become the treatment of choice for patients with stage II–III locally advanced rectal cancer (LARC) [2–6]. Data from a randomized clinical trial performed in 2004 showed that TME preceded by chemoradiation was safer and more effective than TME with no prior treatment in patients with LARC [6–8]. NACRT improves the local control of the malignancy and overall survival, particularly in patients with a complete response to treatment [9, 10]. However, in Europe and the USA, ~ 50% of patients do not receive this treatment, primarily owing to advanced age and comorbidities [6]. Unfortunately, inaccurate pre-operative staging can preclude the possibility for some patients to receive NACRT, particularly those in under-represented ethnic groups, those who present to a low-volume center, and those of low socioeconomic status [6].

It is widely known that NACRT can significantly decrease the number of lymph nodes that are retrieved in surgical specimens [11]. Although this finding has been interpreted by some researchers as being indicative of a good response to NACRT and, therefore, a predictor of positive outcomes, others believe that the retrieval of a lower number of lymph nodes could lead to understaging and stage migration, compromising patient prognosis [3, 5].

In this study, we investigated whether a low number of lymph nodes retrieved in surgical samples after NACRT affects patient outcomes. We also assessed whether the lymph node ratio (LNR)—defined as the ratio of malignant lymph nodes to the total number of lymph nodes retrieved—could be used to accurately determine the cancer stage, and if the LNR is more oncologically meaningful than pathologic evaluation of the pN stage alone.

## Methods

This retrospective analysis included follow-up data from 142 patients with LARC surgically treated at our department between March 2008 and December 2014. The surgery was completed by the same surgeons within one department at three different centers. Follow-up data were prospectively collected at our department until December 2018. This study did not require approval by the Ethics Committee of the University. All patients gave written informed consent to treatment.

During the recruitment period, all 168 patients affected by LARC (based on the pre-surgery staging) were subjected to NACRT followed by TME. Patients treated in the emergency setting, or who had a stage I neoplasia, or exceeded the limits of surgical efficacy were excluded. Patients with symptomatic hepatitis B or C virus infection were also excluded because of known associations with an increased risk of recurrence beyond 5 years, according to personal experience and as reported in the literature [12]. Subsequently to the treatment (NACRT + TME), another 26 patients (15.5% of 168) were excluded from further analysis: 2 cases were lost to peri-operative mortality, 6 cases exhibited advanced intra-operation staging, and 18 patients either chose not to participate or had incomplete follow-up data.

The study inclusion and exclusion criteria are shown in Table 1.

**Table 1:** Patient inclusion and exclusion criteria

Inclusion criteria
Histologically confirmed rectal cancer
Total mesorectal excision with regional lymphadenectomy
Willingness to undergo follow-up
Exclusion criteria
Stage I rectal cancer
Emergency surgery
Presence of synchronous metastases
Cancer invading the adjacent organs
Hepatitis B or C
Absence of complete follow-up data

Therefore, 142 patients divided in two groups were evaluated during the retrospective recruitment period: 71 (50%) patients received neoadjuvant treatment followed by TME (NACRT group) and 71 (50%) patients underwent surgery with no neoadjuvant treatment (no-NACRT group) for age (five patients > 80 years old), comorbidity (15 patients based on the Charlson Comorbidity Index), low socioeconomic status (21 patients who almost all came from the most remote areas of Sicily) or, above all, for their own decision (30 patients).

The treatment protocol for NACRT included induction chemotherapy with the FOLFOX-4 regimen, followed by chemoradiation (45 Gy + 9 Gy and 5-fluorouracil by continuous infusion). The time between the end of NACRT and TME (interval time) was recorded.

For each patient, the distance between the carcinoma and the anus was measured endoscopically and recorded. Cancer was staged according to the 2010 American Joint Committee on Cancer (AJCC) classification criteria [13]: stage 0 (Tis, N0, M0), stage I (T1–T2, N0, M0), stage II (T3–T4b, N0, M0), stage III (T1–T4b, N1–N2b, M0). In the NACRT group, tumor regression after surgery was graded according to the Dworak system [14]: TRG0 (no tumor regression), TRG1 (dominant tumor mass with minor fibrosis: < 25% of the mass), TRG2 (dominant fibrotic changes: 25–60% of the mass, with few tumor cells), TRG3 (fibrotic tissue > 50% of the mass, with very few tumor cells), TRG4 (fibrotic mass with no tumor cells, total tumor regression or response).

For each patient, the number of lymph nodes retrieved, the number of metastatic lymph nodes when present, the percentage of patients with metastatic lymph nodes, and the LNR were recorded. In the NACRT group, only eight patients (11.3%) underwent post-operative chemotherapy. In the no-NACRT group, 24 patients (33.8%) not received post-operative treatment for age, comorbidity or patient rejection; 19 patients (26.8%) received chemoradiation, 18 patients (25.3%) underwent chemotherapy only and 10 patients (14.1%) underwent radiotherapy only.

All patients were followed up every 3 months during the first 3 years after treatment, and then every 6 months during the years 4 and 5. The primary end points were to collect data on the time between the surgery and relapse and the disease-free survival (DFS) rate at 5 years.

### Statistical analyses

Descriptive statistics were used to describe the characteristics of patients in NACRT and no-NACRT groups. Specifically, the Kolmogorov–Smirnov test was used to test the normality of continuous variables. Due to their skewness, all of the continuous variables were reported as the median and interquartile range (IQR) and compared using the Mann–Whitney *U* test. Categorical variables were reported as a percentage and compared using the Chi-squared test. To identify independent determinants of the number of lymph nodes retrieved, a multivariable linear regression analysis was performed. The model included all of the variables that were significantly associated with the number of lymph nodes retrieved in the univariate analysis. A Kaplan–Meier analysis was conducted to evaluate DFS. A receiver operating characteristic (ROC) curve analysis was used to determine whether the number of metastatic lymph nodes and the LNR could be used to discriminate patients who experienced rectal cancer relapse. Accuracy was reported as the area under the curve (AUC) with 95% confidence interval (95%CI) and compared using the Hanley and McNeil method [15]. The optimal cutoff values of the number of metastatic lymph nodes and of LNR maximizing the Youden index were identified. Statistical analyses were performed using SPSS software (version 22.0, SPSS, Chicago, IL). All statistical tests were two-sided, and *P* values < 0.05 were considered statistically significant.

## Results

The median age of the study population was 64.5 years (IQR = 54.0 years); 45 patients (31.5%) were women and 96 (68.5%) were men. The average duration of the follow-up was 47.8 months in the whole study population, 45.3 months in the NACRT group, and 50.3 months in the non-NACRT group (range 12–60 months for all groups).

The median distance between the anus and carcinoma was 7 cm (IQR = 5 cm) in the NACRT group and 10 cm (IQR = 6 cm) in the no-NACRT group. After TME in patients who underwent NACRT, the cancer was staged as follows: 26.8% stage 0; 26.8% stage I; 28.2% stage II; and 18.3% stage III. After TME in the no-NACRT group, 46.5% patients were classified as having stage II cancer and 53.5% with stage III cancer. The degree of tumor regression in the NACRT group was as follows: 15.5% TRG0–1; 59.2% TRG2–3; and 25.3% TRG4. The median number of lymph nodes retrieved was 12 (IQR = 8) in the whole patient population, 10 (IQR = 7.5) in the NACRT group, and 14 (IQR = 8) in the no-NACRT group. Metastatic lymph nodes were found in 36.62% of all patients. Accordingly, 80.3% of patients in the NACRT group did not have metastatic lymph nodes, whereas the proportion was much lower in the no-NACRT group (46.5%). The median LNR was 0 (IQR:1) in the whole patient population, 0 (IQR = 0) in patients in the NACRT group, and 0.06 (IQR:0.24) in the no-NACRT group. The number of lymph nodes retrieved after surgery, the number of metastatic lymph nodes, the LNR and the percentage of patients with metastatic lymph nodes were all significantly lower in the NACRT group than in the no-NACRT group (Table 2).

**Table 2:** Characteristics of patients according to neoadjuvant chemoradiotherapy

Characteristic	No NACRT	NACRT	*P* value^b^
Age (years)^a^	66 (20)	64.0 (12.5)	0.740
Sex (% male)	66.2%	70.4%	0.588
Distance between the tumor and the anus (cm)^a^	10 (6)	7 (5)	** < 0.001**
Number of lymph nodes harvested^a^	14 (8)	10.0 (7.5)	** < 0.001**
Number of metastatic lymph nodes^a^	1 (3)	0 (0)	** < 0.001**
Lymph node ratio^a^	0.06 (0.24)	0 (0)	** < 0.001**
Patients without metastatic lymph nodes (%)	46.5%	80.3%	** < 0.001**
Patients with disease relapse (%)	26.8%	19.7%	0.320
Time to relapse (months)^a^	19 (8)	13 (18)	0.223
Disease-free survival interval (months)^a^	47 (42)	34 (42)	0.548

*NACRT* neoadjuvant chemoradiotherapy

^a^Results are reported as median (interquartile range)

^b^*P* values < 0.05 are reported in bold

Univariate analysis (Table 3) showed that a higher patient age and NACRT were negatively associated with the number of lymph nodes retrieved. By contrast, more lymph nodes were retrieved in patients with a greater distance between the carcinoma and the anus and in those with advanced-stage carcinoma. Patient sex, interval time, and tumor regression grade did not impact the lymph node yield. Multivariate analysis (Table 3) confirmed that only patient age and NACRT affected the number of lymph nodes retrieved, irrespective of the other factors investigated.

**Table 3:** Analyses of the association between characteristics of patients and the number of lymph nodes harvested

Characteristic	*β*	SE	*P* value^a^
*Univariate analysis*			
Age	– 0.174	0.059	**0.004**
Sex (male vs female)	– 1.924	1.295	0.140
Distance between the tumor and the anus	0.527	0.142	** < 0.001**
Interval time	0.207	0.219	0.348
Tumor stage (II–III vs 0–I)	5.583	1.289	** < 0.001**
TRG (4 vs 2–3 vs 0–1)	– 1.416	1.248	0.261
NACRT (yes)	– 5.690	1.116	** < 0.001**
*Multivariate analysis*			
Age	– 0.177	0.055	**0.002**
Distance between the tumor and the anus	0.232	0.165	0.163
Tumor stage (II–III vs 0–I)	2.760	1.890	0.147
NACRT (yes)	– 3.011	1.080	**0.026**

*NACRT* neoadjuvant chemoradiotherapy, *SE* standard error, *TRG* tumor regression grade

^a^*P* values < 0.05 are reported in bold

**Fig. 1:**
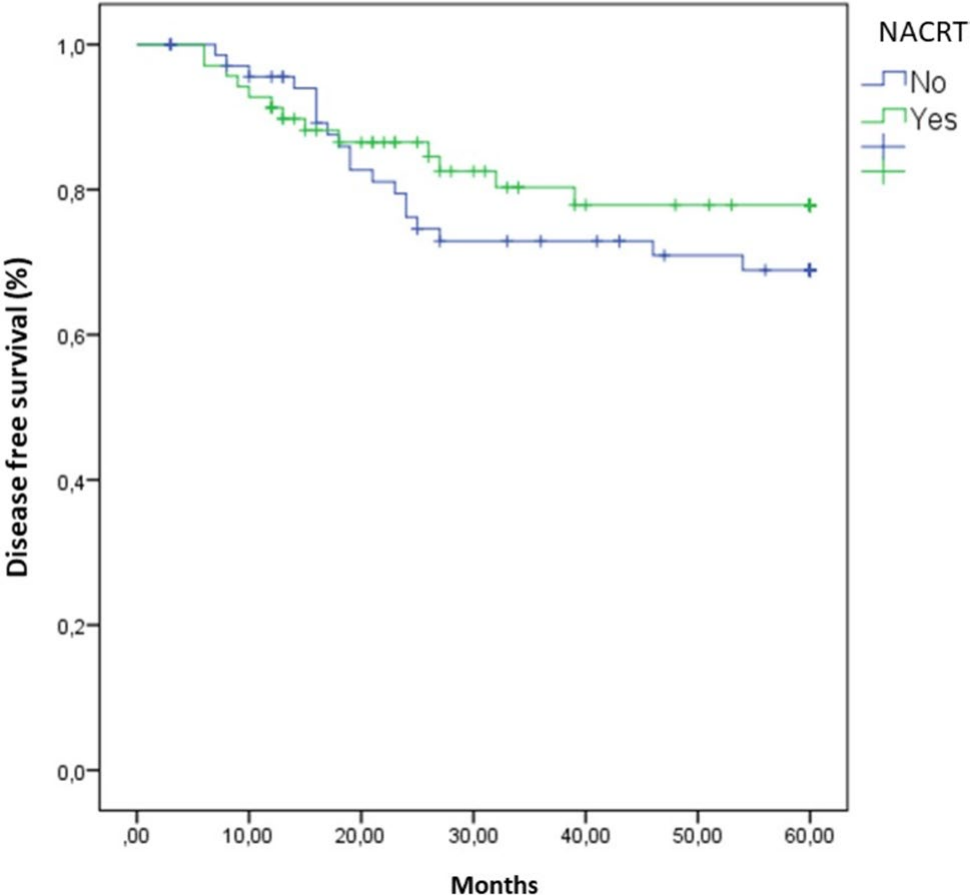
Kaplan–Meier analysis of disease-free survival in patients with locally advanced rectal cancer. *NACRT* neoadjuvant chemoradiotherapy

**Fig. 2:**
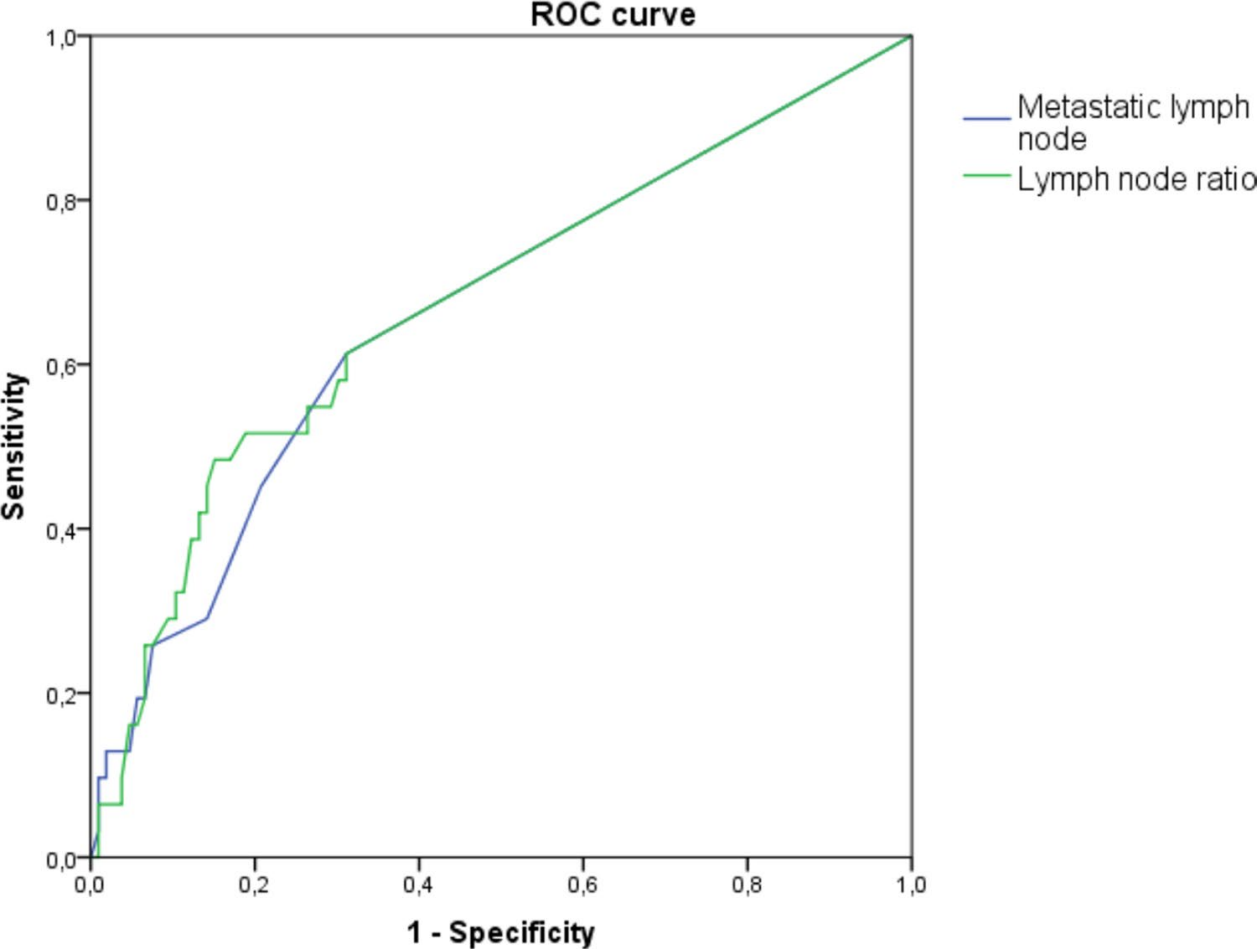
ROC curve analysis of predictors of relapse in patients with locally advanced rectal cancer

Rectal cancer relapse occurred in 23.24% of all patients: 19.7% in the NACRT group and 26.8% in the no-NACRT group. The average interval between the surgery and relapse was 18.9 months in all patients: 17.0 months in the NACRT group and 21.26 months in the no-NACRT group. DFS at 5 years was 80.3% in the NACRT group and 73.2% in the no-NACRT group (Fig. 1). This difference between the two groups was not statistically significant. Across the whole patient population, only the number of metastatic lymph nodes, the LNR, and the percentage of patients with unaffected lymph nodes were significantly associated with risk of relapse (Table 4). Interestingly, the ROC curve analysis demonstrated that the number of metastatic lymph nodes (AUC = 0.663; 95%CI = 0.550–0.777; *p* = 0.006) and the LNR (AUC = 0.674; 95%CI = 0.560–0.789; *p* = 0.003) were able to discriminate patients with relapse, but no difference in their accuracy was evident (*p* = 0.894) (Fig. 2). According to the Youden index, the optimal cutoff values to discriminate patients with rectal carcinoma relapse were one metastatic lymph node and an LNR of 0.19. Consistently, the number of metastatic lymph nodes and the LNR were also indicators of a poor prognosis in the NACRT group (Table 5). However, among patients in the no-NACRT group, only the LNR was significantly associated with rectal cancer relapse (Table 6).

**Table 4:** Characteristics of patients according to prognosis

Characteristic	No relapse	Relapse	*P* value^b^
Age (years)^a^	63 (10)	69.0 (14.5)	0.100
Sex (% male)	68.8%	66.7%	0.817
Distance between the tumor and the anus (cm)^a^	8 (7)	7 (5)	0.492
Patients who received NACRT (%)	47.7%	57.6%	0.320
Number of lymph nodes harvested^a^	13 (7)	13 (9)	0.818
Number of metastatic lymph nodes^a^	0 (1)	1 (4)	**0.003**
Lymph node ratio^a^	0 (0.09)	0.13 (0.32)	**0.001**
Patients without metastatic lymph nodes (%)	69.7%	42.4%	**0.004**
Patients with TRG 4 (%)	25.0%	21.4%	0.780

*NACRT* neoadjuvant chemoradiotherapy, *TRG* tumor regression grade

^a^Results are reported as median (interquartile range)

^b^*P* values < 0.05 are reported in bold

**Table 5:** Characteristics of patients who received NACRT according to prognosis

Characteristic	No relapse	Relapse	*P* value^b^
Age (years)^a^	63 (12)	65.9 (19.5)	0.650
Sex (% male)	70.2%	71.4%	0.927
Distance between the tumor and the anus (cm)^a^	6 (7)	7.0 (3.8)	0.186
Number of lymph nodes harvested^a^	9 (9)	13.0 (9.5)	0.373
Number of metastatic lymph nodes^a^	0 (1)	0.5 (3.0)	**0.018**
Lymph node ratio^a^	0 (0.09)	0.04 (0.27)	**0.007**
Patients without metastatic lymph nodes (%)	57.9%	35.7%	0.136

*NACRT* neoadjuvant chemoradiotherapy

^a^Results are reported as median (interquartile range)

^b^*P* values < 0.05 are reported in bold

**Table 6:** Characteristics of patients who did not receive NACRT according to prognosis

Characteristic	No relapse	Relapse	*P* value^b^
Age (years)^a^	63 (20)	73.9 (13.0)	0.153
Sex (% male)	67.3%	63.2%	0.927
Distance between the tumor and the anus (cm)^a^	11.5 (7.8)	9 (5)	0.429
Number of lymph nodes harvested^a^	14 (9)	14 (9)	0.286
Number of metastatic lymph nodes^a^	0 (2)	2 (4)	0.066
Lymph node ratio^a^	0 (0.16)	0.22 (0.40)	**0.040**
Patients without metastatic lymph nodes (%)	51.9%	31.6%	0.128

*NACRT* neoadjuvant chemoradiotherapy

^a^Results are reported as median (interquartile range)

^b^*P* values < 0.05 are reported in bold

## Discussion

An accurate pathologic assessment of lymph nodes is crucial to ensure correct LARC staging and management, and it is the strongest predictor of long-term outcomes [3, 4, 11, 16]. The AJCC recommends that at least 12 lymph nodes are retrieved to establish patient prognosis [13]. However, the number of lymph nodes typically retrieved can vary greatly ranging from 6 to 13 [15], and is < 12 for 30–50% of patients [1, 3, 18–20]. A small number of retrieved lymph nodes can lead to understaging, suboptimal treatment, and consequently a poor prognosis [3–5, 11, 21, 22]. Unfortunately, a numerically adequate lymph node yield is often impossible to achieve owing to ‘modifiable’ and ‘non-modifiable’ factors related to the surgeon (e.g.. experience, specialization), the pathologist (e.g., diligence of pathology staff, time factors), the patient (e.g,. age, sex, obesity), and the disease (e.g., cancer stage, tumor site and distance from the anus) [11].

In our analyses, patient sex, the distance between the tumor and the anus, interval time, tumor stage, and tumor regression grade did not affect the number of lymph nodes retrieved, whereas patient age and NACRT significantly reduced the number of lymph nodes in surgical samples (Table 3). No consensus on the effects of these parameters on lymph node sampling exists in the literature [3, 5, 11].

Lymphoid mesorectal tissue is extremely radiosensitive, and NACRT can cause apoptosis, stromal atrophy, and fibrosis, leading to a low number lymph nodes available for retrieval [3–5, 16, 19, 22, 23]. Indeed, the number of lymph nodes available for retrieval in this contest is reportedly < 12 in one out of three patients [3, 19]. The lower number of lymph nodes retrieved after NACRT from the patients included in our study compared with the number retrieved in patients who did not receive NACRT could indicate an improved response to treatment rather than inadequate surgical resection and/or pathologic examination. Moreover, fewer lymph nodes in a surgical sample could be used as a marker for favorable tumor behavior and, consequently, a good prognosis [3–5, 19, 20, 22, 24].

We also found that patients in the NACRT group had significantly fewer metastatic lymph nodes than patients in the no-NACRT group. Consensus in the literature varies in terms of the relationship between the number of metastatic lymph nodes and prognosis [3, 4, 25]. In our study, patients who underwent NACRT tended to have a better (although not significantly so) 5-year DFS (80.3% versus 73.2%) (Fig. 1). The total number of lymph nodes retrieved and the total number of metastatic lymph nodes were lower in this group (Table 2). However, neither the total number of lymph nodes nor NACRT significantly affected patient prognosis (Table 4). By contrast, the presence of metastatic lymph nodes, which are considered a marker of tumor aggression [3], did adversely affect patient outcomes (Table 4). Therefore, we postulate that NACRT only influences prognosis when it reduces the number of metastatic lymph nodes.

Pre-operative chemoradiation reduces the tumor size and results in down-grading of lymph node stage. Some researchers, therefore, suggest that a reduced lymph node yield is associated with overall tumor pathological regression [3, 5]. In this study, we detected a trend toward better survival in patients with TRG4 (Table 4). This trend might have been statistically significant if our sample had been larger. Nevertheless, the greater degree of tumor regression in this group did not affect the number of lymph nodes retrieved (Table 3). Although reports in the literature vary [20, 26, 27], our results concur with those of Loftas et al. [28] and Shwaartz et al. [29] who found that a complete pathological response does not exclude the presence of metastatic lymph nodes. These findings raise questions about the ‘watch and wait’ management strategy for patients with seemingly complete tumor regression [30].

In patients with stage II cancer after NACRT, retrieving at least 12 lymph nodes is essential to certify node-negative status, and to prevent understaging or stage migration. In this respect, a literature review published by the Italian Society of Surgical Oncology Colorectal Cancer Network (SICO-CNN) produced some interesting results [27]. Among 1407 patients treated with NACRT and staged ypN0 (stage II), the number of lymph nodes retrieved, even when < 12, was not associated with survival and did not, therefore, have prognostic value. By contrast, among patients with metastatic lymph nodes (stage III), the LNR had a prognostic role regardless of the lymph node yield [11, 31]. In our opinion, if the LNR were incorporated into the AJCC staging system [13] when the lymph node yield is < 12, the incidence of stage migration would be reduced [17].

To our knowledge, this analysis is the first to show that both the number of metastatic lymph nodes and the LNR are significantly associated with patient prognosis (Table 4), and that the two parameters are equally prognostic for local relapse (Fig. 2). When we evaluated the two parameters independently, we found that the LNR could significantly predict patient outcomes in both treatment groups, whereas the number of metastatic lymph nodes was a predictive factor only among patients who underwent NACRT (Tables 5, 6). A consensus on the LNR cutoff, which would allow us to reliably predict which patients are most likely to experience relapse [16, 17, 19, 22, 32], has not yet been established. In our previous study of patients with stage II–III colorectal cancer, we found that the 5-year DFS was 71% in patients with an LNR $\leq$ 0.194 and 45% those with an LNR $\ge$ 0.194 (log-rank test, *P* < 0.001) [31]. This result indicates that an LNR cutoff of 0.194 would be appropriate.

## Conclusions

In light of our results and other data in the literature [2, 4, 5, 16, 19, 21–23, 32–34], we believe that the LNR is a better parameter than pN stage for (i) predicting the risk of LARC relapse and for (ii) accurately staging LARC. New mathematical models for lymph node staging are currently being tested. For example, data thus far suggest that the LODDS (the logarithm of the ratio between the number of positive nodes and the number of negative nodes) might be more effective for staging than the LNR, although further randomized clinical trials are needed to refine stratification and improve prediction of survival in patients with LARC [1, 17].

Although our study was limited by its retrospective design, the data were collected prospectively. In addition, a larger sample of patients might have generated more significant results.

The main drawback of our study was that in the no-NACRT group, the post-operative treatment different among the patients, which is why we could not consider this variable in the patient’s prognostic evaluation. Moreover, due to the retrospective nature of the study, we could not determine the limit of the Charlson Comorbidity Index over which a patient would be excluded from receiving neoadjuvant treatment. We were also unable to accurately determine the exact difference between the variables “socioeconomic status” and “personal patient decision” to justify the exclusion of some patients from the neoadjuvant treatment.

Nevertheless, we believe that lymph node sampling remains an indispensable tool for staging and prognostic assessment of patients with rectal carcinoma treated with NACRT. Going forward, we think that the focus should move away sampling a minimum of 12 lymph nodes, and move toward a thorough assessment of possible metastatic lymph nodes, even in patients with an apparent complete response to neoadjuvant therapy (TRG4). We believe that NACRT only improves prognosis when the number of metastatic lymph nodes is reduced, and the LNR should be considered the most important predictor of patient outcomes.
